# Lights Off, Lights On: Amaurosis Fugax in Polycythemia

**DOI:** 10.7759/cureus.25752

**Published:** 2022-06-08

**Authors:** Shao Sze Tan, Amir Samsudin, Lakana Kumar Thavaratnam, Masnon Nurul-Ain

**Affiliations:** 1 Ophthalmology, Hospital Seremban, Seremban, MYS; 2 Ophthalmology, University of Malaya, Kuala Lumpur, MYS; 3 Ophthalmology, University of Malaya Medical Centre, Kuala Lumpur, MYS; 4 Ophthalmology, Sunway Eye Centre, Kuala Lumpur, MYS; 5 Ophthalmology, Hospital Kuala Lumpur, Kuala Lumpur, MYS

**Keywords:** polycythemia rubra vera, polycythemia vera, venesection, polycythemia, amaurosis fugax

## Abstract

There are many causes of amaurosis fugax, including polycythemia. Polycythemia is associated with elevated hematocrit levels and hyperviscosity, which can lead to ocular manifestations. We report a polycythemia patient with amaurosis fugax, who had resolution of ocular symptoms following venesection. A 29-year-old gentleman presented with a six-month history of episodic bilateral transient loss of vision (amaurosis fugax), followed by slow recovery back to normal after 15-20 minutes. The symptoms worsened with fatigue. He also had an unsteady gait for the preceding one year. Ocular examination was unremarkable. His visual acuity was 20/20 OU. Neurological examination revealed gait ataxia and dysdiadochokinesia. Computed tomography (CT) angiogram showed an old cerebellar infarct. Blood investigations showed persistent elevated hemoglobin and hematocrit with positive JAK-2 V617F mutation. Infective and connective tissue workups were all negative. A diagnosis of polycythemia was made by the haematology team. In addition to oral aspirin given by the neurology team, he underwent venesection with improvement in ocular symptoms following each episode of venesection. The frequency of amaurosis fugax reduced from 2-3 episodes a week to once a month, then resolved completely after five venesections. Systemically, his cerebellar symptoms also resolved and there were no neurological deficits. Polycythemia is a rare disease that can cause amaurosis fugax and thrombotic events in young patients. Better knowledge and accurate diagnosis are important, as early treatment may improve the symptoms and long-term morbidity.

## Introduction

Amaurosis fugax (AF) is defined as a sudden and transient vision loss, typically lasting from seconds to minutes with complete recovery to normal in between attacks [1]. Unilateral presentation is more common, mostly attributed to vascular causes including ipsilateral carotid artery disease with thromboembolism, and giant cell arteritis. Although less common, bilateral cases may occur in conditions such as vertebrobasilar ischemia, bilateral carotid disease, migraine or seizures. AF may also occur in hypercoagulable states, especially in systemic lupus erythematosus (SLE), antiphospholipid syndrome and thrombophilia. AF in polycythemia is uncommon. We report a rare case of AF as an early manifestation of polycythemia in a young male patient.

## Case presentation

A 29-year-old non-smoker gentleman presented with a six-month history of bilateral transient loss of vision (amaurosis fugax) occurring two to three times a week. Each episode would appear suddenly, last for 15 to 20 minutes, and then recover back to normal. These episodes worsened when he was tired. He also complained of generalised lethargy and occasional dizziness. He also had an unsteady gait for the preceding one year.

Ocular examination showed visual acuity 20/20 OU. Anterior and posterior segment examination was unremarkable. There were no retinal ischemia, retinal haemorrhages, or Hollenhorst plaques. His blood pressure was 135/80mmHg, pulse rate was 80 beats per minute, regular and his fasting blood sugar level was 4.8mmol/L. He was alert and well hydrated with no hepatosplenomegaly. Neurological examination revealed gait ataxia and dysdiadochokinesia. Other systemic examinations were unremarkable.

Contrast-enhanced computed tomography (CECT) of the brain showed an old cerebellar infarct. Electrocardiogram (ECG), echocardiogram and ultrasound carotid Doppler showed normal results. His investigation results were as in Table 1. Full blood count revealed elevated hemoglobin levels of 17.8 g/dL and hematocrit of 0.55 (55%) with normal white blood cell and platelet counts. Erythrocyte sedimentation rate (ESR), C-reactive protein (CRP), thyroid function, screening for rapid plasma regain, hepatitis and retroviral infections were normal. Connective tissue and vasculitis workup revealed normal complement level, negative anti-nuclear antibody test (ANA), anti-neutrophil cytoplasmic antibody (ANCA) and rheumatoid factor. Anti-cardiolipin antibodies were negative. His fasting blood sugar and cholesterol were within the normal limit. JAK-2 V617F mutation testing for polycythemia was positive. He was diagnosed as having polycythemia.

**Table 1:** Laboratory findings Units: g/dL, grams per deciliter; fL, femtoliter; pg, picogram; mmol/L, millimoles per liter; µmol/L, micromoles per liter; U/L, units per liter; mIU/L, milli-international units per liter; pmol/L, picomoles per liter

Investigation	Result	Reference range
Full blood count		
Hemoglobin	17.8 g/dL	13.0-17.0
Hematocrit	55%	40-50
White blood cell	6.41 x 10^9^/L	4.00-11.00
Platelet	341 x 10^9^/L	150-410
Mean cell volume	80.5 fL	83.0-101.0
Mean cell hemoglobin	26.4 pg	27.0-32.0
Mean cell hemoglobin concentration	32.8 g/dL	31.5-34.5
Red cell distribution width	15.6%	11.6-14.0
Neutrophil	4.11 x 10^9^/L	2.0-7.0
Neutrophil %	64.1%	40.0-80.0
Lymphocyte	1.69 x 10^9^/L	1.0-3.0
Lymphocyte %	26.4%	20.0-40.0
Monocyte	0.48 x 10^9^/L	0.2-1.0
Monocyte %	7.5%	2.0-10.0
Eosinophil	0.11 x 10^9^/L	0.02-0.50
Eosinophil %	1.7%	1.0-6.0
Basophil	0.02 x 10^9^/L	0.02-0.10
Basophil %	0.3%	<2.0
Urea	5.0 mmol/L	2.8-8.1
Sodium	140 mmol/L	136-145
Potassium	4.0 mmol/L	3.4-4.5
Chloride	105 mmol/L	98-107
Creatinine	91 µmol/L	62-106
Total protein	76 g/L	64-83
Albumin	39 g/L	35-52
Globulin	37 g/L	
A/G ratio	1.1	
Total bilirubin	8 µmol/L	0-21
Alkaline phosphatase	77 U/L	40-130
Alkaline aminotransferase	22 U/L	0-41
HbA1c (glycated hemoglobin)	5.3%	<6.5
Fasting blood sugar	5.3 mmol/L	3.0-6.1
Total cholesterol	5.3 mmol/L	<5.2
HDL (high-density lipoprotein) cholesterol	1.2 mmol/L	>1.6
LDL (low-density lipoprotein) cholesterol	3.3 mmol/L	<2.6
Triglycerides	1.8 mmol/L	<1.7
Total calcium	2.37 mmol/L	2.15-2.50
Magnesium	0.87 mmol/L	0.66-1.07
Phosphate (inorganic)	0.87 mmol/L	0.81-1.45
Thyroid-stimulating hormone (TSH)	0.66 mIU/L	0.27-4.20
Free thyroxine T4	14.56 pmol/L	12.00-22.00
Rapid plasma reagin (RPR)	Negative	
Hepatitis B	Negative	
Hepatitis C	Negative	
Retroviral (HIV)	Negative	
Antinuclear antibody (ANA)	Negative	
Anti-neutrophil cytoplasmic antibody (ANCA)	Negative	
Rheumatoid factor (RF)	Negative	
Anti-cardiolipin antibodies	Negative	
Anti-beta-2-glycoprotein	Negative	
JAK-2 V617F mutation	Positive	

The patient was started on oral aspirin for the cerebellar infarct by the neurology team and was arranged to have serial venesection by the haematology team. He underwent venesection every four-monthly, with each venesection lasting 15 to 30 minutes and where approximately 500ml of blood was removed. Following each episode of venesection, he reported improvement in ocular symptoms. The frequency of transient vision loss reduced from two to three episodes per week, to once a month following the first two episodes of venesection. After a total of five venesections, he did not report any more episodes of amaurosis fugax. On the latest follow-up after three years, he remained stable with no further episodes of amaurosis fugax or recurrence of cerebellar stroke. Bilateral visual acuity remained 20/20 and his cerebellar symptoms had resolved with physiotherapy and occupational therapy.

## Discussion

Polycythemia is a form of myeloproliferative neoplasm where there is erythrocytosis and increased hematocrit, along with the increased release of inflammatory cytokines [2]. Patients with polycythemia have a higher risk of developing thrombosis, even in young individuals [3]. Polycythemia is rarely encountered in amaurosis fugax. The incidence of polycythemia is higher among males compared to females, which commonly occurs around the age of 60 years [4].

There are various aetiologies of amaurosis fugax. These include those that require early intervention and treatment, such as giant cell arteritis, cardioembolism and ocular ischaemic syndrome [5]. Systemic diseases are also often associated with amaurosis fugax. These include heritable thrombophilia [6-7], Trousseau syndrome [8] and antiphospholipid syndrome [9]. In our patient, systemic risk factors for thrombotic events in young patients including systemic lupus erythematosus, young hypertension, diabetes mellitus, anti-phospholipid syndrome, thrombophilia, familial hyperlipidemia and Moyamoya disease had been ruled out. Neurology conditions that may mimic amaurosis fugax including seizure and migraine had also been ruled out.

Ocular symptoms and signs in polycythemia usually occur mainly as a result of hyperviscosity from elevated hematocrit levels [10]. It has been reported that during episodes of retinal migraine and amaurosis fugax, fundus fluorescein angiography performed during that time can show delayed filling of the central retinal vessels [11]. In severe cases, cilioretinal artery occlusion may occur in polycythemia [12] which can lead to irreversible blindness. Systemic manifestations of polycythemia include fatigue, abdominal discomfort, bone pain, sexual dysfunction, itching and blurring of vision; while some of these symptoms may be mild, they can affect the quality of life [13]. Patients with polycythemia have a higher risk of developing thrombosis, especially myocardial infarction or stroke [3]. In our case, our patient suffered from cerebellar stroke. From an analysis of the REVEAL study, the four-year mortality rate among polycythemia patients was estimated to be more than 10% with various causes of death, with one-third being from thrombotic complications [14]. Progression to myelofibrosis or transformation to acute leukemia resulted in significant mortality.

The current management in polycythemia aims for hematocrit levels <45%, which has been associated with reduced thromboembolic events [15]. Risk stratification often classifies patients into low-risk or high-risk categories. For low-risk categories including our patient, treatment options include low-dose aspirin and venesection. The benefits of venesection in polycythemia include haemodilution, with increased oxygen tension and decreased carbon dioxide tension in arterial blood; additionally, there is also improved arterial flow velocity [16]. Patients with higher risk of developing thrombosis can be started on hydroxyurea or interferon-α as first-line therapy, along with JAK-2 inhibitors if refractory [17].

## Conclusions

Identification and recognition of polycythemia is important, as it is a treatable cause of amaurosis fugax. Without appropriate treatment, patients are at risk of thrombotic events. The disease may also progress to myelofibrosis and acute leukemia. Early diagnosis may reduce patients’ morbidity and mortality.
